# Development and validation of spectrophotometric method and paper-based microfluidic devices for the quantitative determination of Amoxicillin in pure form and pharmaceutical formulations

**DOI:** 10.1016/j.heliyon.2024.e24968

**Published:** 2024-01-22

**Authors:** Jwan Oday, Hind Hadi, Parween Hashim, Samantha Richardson, Alexander Iles, Nicole Pamme

**Affiliations:** a Department of Chemistry, College of Science, Mustansiriyah University, Baghdad, Iraq; b Department of Chemistry, College of Science, University of Baghdad, Baghdad, Iraq; c Department of Chemistry, College of Science, University of Duhok, Duhok, Iraq; d School of Natural Sciences, University of Hull, Cottingham Road, Hull, HU6 7RX, UK; e Department of Materials and Environmental Chemistry, Stockholm University, 106 91, Stockholm, Sweden

**Keywords:** Amoxicillin (AMX), Spectrophotometry, Diazotized sulfadimidine (DSDM), Paper-based microfluidic device, Pharmaceutical dosage forms

## Abstract

There is a growing need for easy-to-use, low cost and portable quantitative assays to determine active pharmaceutical ingredients in the pharmaceutical industry. Here, we developed a batch spectrophotometric method and a method employing a paper-based microfluidic device for the estimation of Amoxicillin (AMX) in pure solution and pharmaceutical preparations. The detection depends on the coupling reaction of Amoxicillin with diazotized sulfadimidine (DSDM) in an alkaline medium. The yellow azo dye reaction product was measured at *λ*_max_ 425 nm and linearity was observed from 2 to 30 mg L^−1^ with a detection limit of 0.32 mg L^−1^ and a quantification limit of 1.2 mg L^−1^ was found. The reaction was then transferred onto the paper-based microfluidic device and a plateau change in color intensity was found above 10 mg L^−1^. Thus, the paper-based microfluidic device can be applied for the semi-quantitative determination of Amoxicillin in pure solution and commercial pharmaceutical products for rapid screening.

## 1 Introduction

Constant design and development of pharmaceuticals are vital for the prevention and treatment of diseases. Key parts of this development process include ensuring purity during quality control and the analysis of the active pharmaceutical ingredients (APIs) [1]. Most of the techniques used [2] require relatively sophisticated instruments, costly reagents or complex, multistep sample preparation [3,4]. In low- and middle-income countries issues of poor quality pharmaceuticals persist [5]. The limited resources in such environments require that an analytical method has minimal costs for the equipment as well as consumables to ensure pharmaceuticals are fit for purpose [6]. Therefore, there is an essential need for the development of deployed and rapid analytical techniques to achieve this goal [7].

In recent years, various analytical applications of paper-based microfluidic devices to perform chemical analysis have gained great interest due to their many advantages, including being low cost, portable and having a low consumption of reagents in comparison to other analytical techniques [8]. A particular area of growth has been in the application of paper-based microfluidic devices for qualitative and quantitative determination of pharmaceuticals which has recently been reviewed by Sharma et al., concluding that such devices have much potential as screening tools within the pharmaceutical industry [9]. Noviana et al. concluded that paper-based systems do not have the accuracy or precision of traditional instrumentation [10]. However, they do offer an alternative low-cost, rapid screening tool for resource-limited settings to facilitate screening, where it may not exist currently at all.

Amoxicillin (6-(p-hydroxy-alpha-amino phenyl acetamido) penicillanic acid) belongs to the aminopenicillin group penicillin antibiotic [11]. It is a white powder with sulphureous odor and has a water solubility of 958 mg mL^−1^ [12]. Amoxicillin is used to treat various types of bacterial infections, for example pneumonia, pneumonia, and urinary tract infection, since it has the capability to inhibit the synthesis of bacterial cell walls [13]. Spectrophotometric [[14], [15], [16], [17]], chromatographic [[18], [19], [20]] and electrochemical [21,22] techniques are among several methods reported for determining this drug in pharmaceutical preparations. Whilst current techniques are highly accurate and sensitive in general they fail to achieve the criteria of being low cost, rapid, or easy to operate that are required for high throughput screening [23].

Here, we target these challenges by studying a Amoxicillin trihydrate (AMX) via colorimetric assays since they are portable, low cost and can be used without the need for expensive detection equipment [24]. In the present paper, the coupling reaction between AMX and diazotized sulfadimidine (DSDM) in alkaline medium yielding a colored azo dye product with spectroscopic and paper-based microfluidic readout was investigated to develop a rapid, simple analytical platforms for detection of AMX as active ingredients in drug formulations.

## 2 Experimental

### 2.1 Equipment and materials

All spectral scans and measurements were done using a Shimadzu UV–Visible 1240 digital single-beam spectrophotometer. Throughout this study, analytical reagent grade chemicals were used. Pure AMX was kindly provided by State Company for Drug Industries and Medical Appliance (SDI-Samara/Iraq). Pharmaceutical applications (Amoxicillin sodium® 500 mg injection-PYXUS/India) were purchased from a local pharmacy in Baghdad, Iraq.

### 2.2 Preparation of solutions

**Amoxicillin sodium (AMX):** Stock solutions of 250 and 500 mg L^−1^ were obtained by weighing 0.0250 and 0.0500 g of AMX pure drug, respectively, and making up to 100 mL with deionized water. Serial dilutions were carried out with deionized water to prepare the working standard solutions. **Sodium hydroxide solution (NaOH):** 2 M of sodium hydroxide solution was made from 20 g of NaOH (Merck) dissolved in 250 mL deionized water. Working solutions were obtained by serial dilution with deionized water. **Diazotized sulfadimidine (DSDM):** A 10 mM solution was made by the addition of 0.9 mL standard sulfadimidine sodium solution (333 mg L^−1^) to a 100 mL volumetric flask and 3 mL of 1 M HCl (36 % w/w) in an ice–bath. This was left to incubate for 5 min afterwards, sodium nitrite (0.0690 g) was added to the mixture and made up to volume with deionized water.

### 2.3 Analysis of commercial dosage forms

**Solution of AMX dosage form:** Five vials of Amoxicillin® injections were combined. From these, an aliquot corresponding to 50 mg of the drug was diluted to 100 mL with deionized water to obtain a final concentration of 500 mg L^−1^. Prior to analysis, the formulation was adjusted to pH 12.4 using sodium hydroxide to mimic conditions used for the standards.

### 2.4 Spectrophotometric detection of AMX

**For AMX:** A calibration curve was constructed by plotting the absorbance of the AMX working solution in the range of (2, 4, 6, 10, 15, 20, 25, 30) mg L^−1^ prepared by serial dilution of the 500 μg mL^−1^ stock solution into 10 mL volumetric flasks. Then, both 2.5 mL of DSDM (10 mM) and 0.25 mL of NaOH (0.5 M) were added. Next deionized water was used to dilute the solutions to the mark, mixed well and left for 10 min at room temperature (25 °C). The absorbance was measured at 425 nm versus the reagent blanks containing all above reagents except for AMX. For all optimization experiments, 25 mg L^−1^ of AMX was employed. Three repeats were carried out for each of the optimization and calibration experiments, by taking three readings for each individual set of parameters. The standard deviation of the three readings were used to calculate the error bars.

### 2.5 Paper based microfluidic platform detection of AMX

**Paper-device fabrication:** The microfluidic paper-based analytical devices was designed based on Carrilho et al. method with AutoCAD 2016 software and using a wax printer (Xerox ColorQube 8570) and wax inks (ColorQube). Designs were printed onto Whatman Grade 1 filter paper [25]. Akin to a method from Peters et al. [26], a hydrophobic barrier was made to penetrate the depth of the paper by passing the wax-printed sheets through a Fellows, Model Callisto A4 laminator at 125 °C three times. This allowed the wax to melt through the porous paper. Our design included eight circular features, with an 8 mm center to center distance and a 10 mm diameter. The lines were printed with a width of 0.7 mm in green wax (Fig. 5). A set of six colored squares as an internal standard was included in each device to account for camera-to-camera differences and differences in lighting conditions during the image analysis.

**Assay measurements:** The eight reaction circles of the microfluidic paper device would allow for the measurement of eight separate standard concentrations of AMX on the same device. The on-paper reaction was carried out by separately spotting 5 μL of the different standard solutions of AMX into seven of the reaction circles. These were then left to dry. The eighth circle was left empty and used to calculate the blank. Next, 10 μL of the reagent DSDM (5 μL) as well as NaOH (5 μL) were pipetted into each reaction circle. The mixture was left to dry before a photograph was taken with the camera of a Samsung Note 9 smartphone. The pharmaceutical preparations were detected using the same procedure. Three repeats for carried out for each of the optimization and calibration experiments. Error bars were calculated using the standard deviation of the three readings.

**Image analysis***:* For image analysis, Image J freeware (National Institutes of Health, USA) was used (Fig. 6) following a published method by Boehle et al. [27]. Firstly, the transferred jpg images were inverted. Then images were split into three channels, RED, GREEN and BLUE. The BLUE channel was selected for AMX reaction since it featured the highest intensity difference for varying sample concentrations. We then employed the mean grey pixel intensity to interrogate the area within the green wax circle to obtain an average pixel intensity for each of the reaction circles. The colored reference squares included in the microfluidic design we employed in order to reduce the impact of differences in lighting conditions. For each individual circle, the average pixel intensity (AI) was divided by the average pixel intensity of the yellow reference square to obtain the average relative intensity (ARI) (equation (1)).

(Eq 1) $$Avereage\;relative\;intensity\;\left(ARI\right)=\frac{Average\;intenisty\;of\;each\;reaction\;zone}{Intenisty\;of\;refernce\;square}$$

## 3 Results and discussion

### 3.1 Spectrophotometric analysis for AMX measurements

**Fig. 1:**
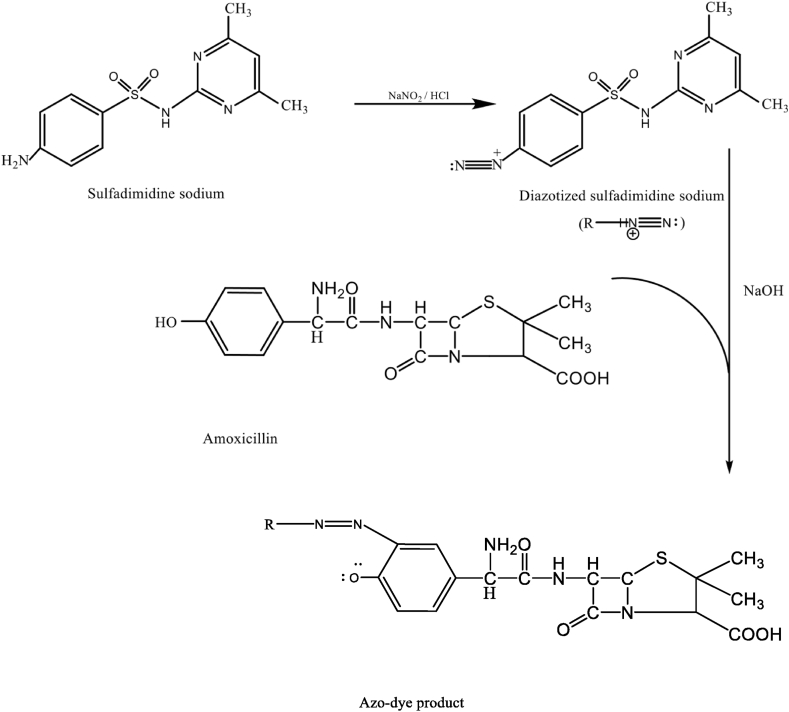
Reaction scheme between AMX with DSDM in alkaline medium.

**Fig. 2:**
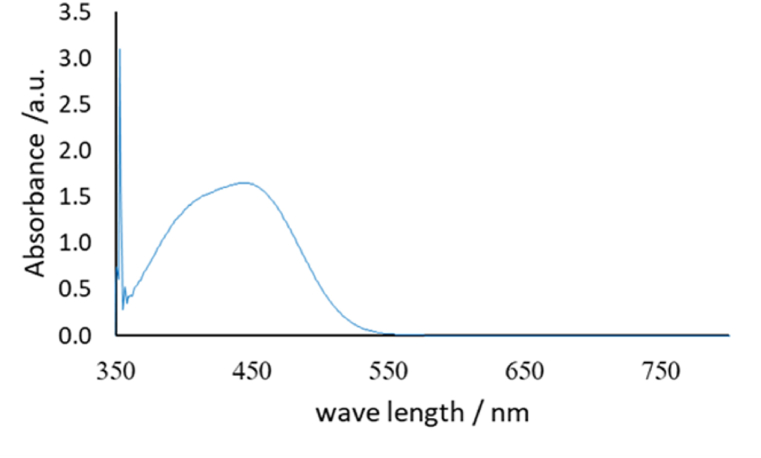
Absorption spectrum of the yellow dye produced from the reaction of AMX (25 mg L^−1^) with DSDM in an alkaline medium. (For interpretation of the references to color in this figure legend, the reader is referred to the Web version of this article.)

The coupling reaction between AMX and DSDM leads to a colored product as shown in Fig. 1. An immediate formation of a yellow azo dye was observed after the addition of DSDM solution to AMX under alkaline conditions. The yellow product was found to have an absorbance maximum of 425 nm (Fig. 2).

**Fig. 3:**
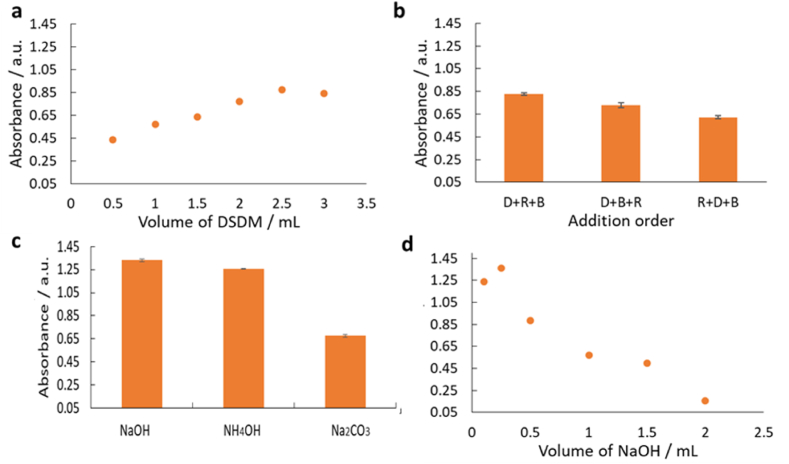
Effect of different reaction parameters on the formation of the azo dye. All experimental parameters were optimized using 0.5 mL of 25 mg L^−1^ AMX solution, n = 3. (a) Volume DSDM (10 mM) varied between 0.5 mL and 3.0 mL, RSD ≤1 %. (b) Reagent addition order (D = drug, R = reagent, B = base) to determine optimum reaction conditions. (c) Varying the base used to produce the required alkaline conditions for the coupling of AMX with DSDM to form azo-dye products. Different bases (NaOH, NH_4_OH, Na_2_CO_3_), all at 0.5 M, were investigated. (d) Volume of NaOH (0.5 M) varied between 0.1 mL and 2 mL, RSD ≤2 %.

The dye-formation reaction was first optimized with absorbance measured via the UV/vis spectrometer at 425 nm relative to a blank solution. For optimization, 25 mg L^−1^ of AMX was employed. Initially, the type and volume of the base solution were investigated by reacting 0.5 mL of the drug solution with 2.5 mL reagent. At basic pH above 12.4, AMX forms phenoxide anions, which readily react with DSDM leading to the azo-dye formation. When varying the volume of DSDM reagent (10 mM) in the range of 0.5 mL–3.0 mL (Fig. 3a), 2.5 mL was adequate for the maximum absorbance. The reaction is a two-step process requiring a change in pH from the intermediate step one to step two. Therefore the mixing order of AMX, DSDM and base was also varied and it was found that the drug (D) followed by the reagent (R) DSDM and then the base (B) gave the strongest response (Fig. 3b). When comparing 0.5 M NaOH, NH_4_OH and Na_2_CO_3_ (Fig. 3c) it was found that sodium hydroxide gave the maximum absorbance. The volume of NaOH was optimized as shown in (Fig. 3d) giving the highest color intensity for 0.25 mL.

**Fig. 4:**
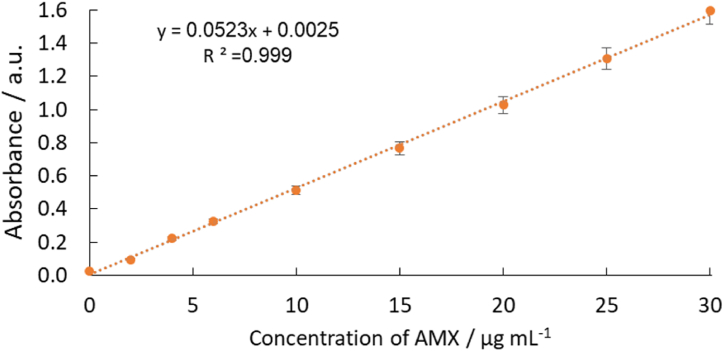
Calibration graph obtained for AMX (0–30 mg L^−1^), n = 4, error bars represent SD, RSD ≤3 %.

For quantitative analysis of AMX, a calibration curve over the range of 2–30 mg L^−1^ was obtained under the optimized parameters (Fig. 4). The respective limits of quantification and limits of detection were calculated and found to be 1.2 and 0.37 mg L^−1^.

**Fig. 5:**
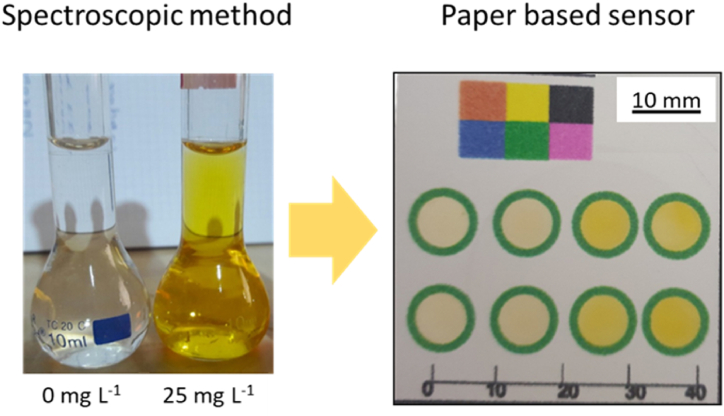
Switching from traditional batch spectroscopic method to paper-based microfluidic platform.

**Fig. 6:**
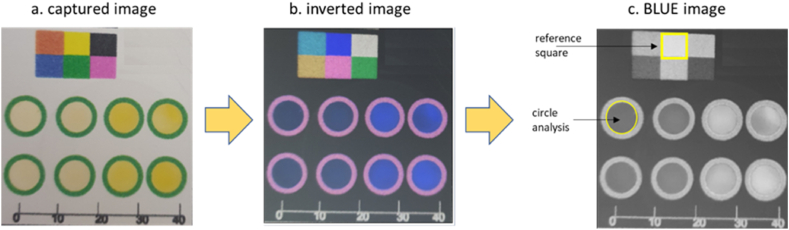
Process of image analysis (a) images captured using Samsung note9 camera (b) inversion and splitting of these images into three channels (RED, GREEN and BLUE), and (c) color intensity measurements using the blue channel. (For interpretation of the references to color in this figure legend, the reader is referred to the Web version of this article.)

### 3.2 AMX measurements via paper-based microfluidic device

Paper-based microfluidic devices were investigated as an alternative to carry out analysis in a laboratory-independent manner. Paper devices rely on capillary action to manipulate fluids [28], the paper cellulose fibers constitute a porous matrix. The azo dye complexes are confined inside the wax printed circular detection zones. The aqueous reagents were pipetted inside the circular wax region (Fig. 5).

Image analysis of the paper devices was undertaken using Image J software by inverting the captured image and splitting each image into individual color channels (RED, GREEN, BLUE). For each analyte a single channel was analyzed, for AMX which produced a yellow complex the BLUE channel was used (Fig. 6(a–c)).

**Fig. 7:**
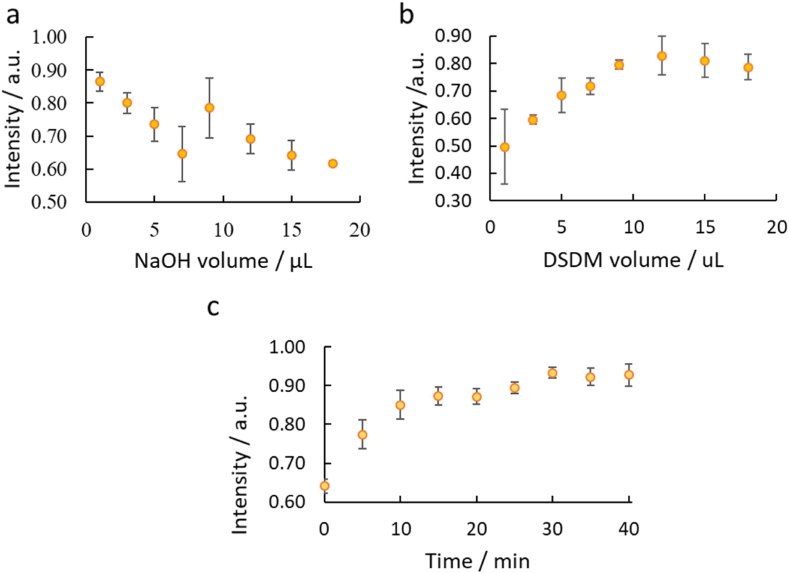
Effect of different reaction parameters on the formation of the yellow azo dye. All experimental parameters were optimized using 25 mg L^−1^ AMX solution, n = 3. **(a)** Volume of NaOH (0.5 M) varied between 1 μL and 18 μL. **(b)** The volume of DSDM reagent (10 mM) varied between 1 μL and 18 μL. **(c)** Development of yellow azo-dye product over time using a 25 mg L^−1^ AMX solution, absorbance recorded between 0 and 40 min every 5 min, n = 4. (For interpretation of the references to color in this figure legend, the reader is referred to the Web version of this article.)

Various parameters were studies to optimize the maximum color intensity on the paper-based microfluidic devices. The effect of different volumes of base and diazotized reagents was studied. A decrease in intensity maybe caused by insufficient reagent volumes, while a reagent leakage out of the sensing zone would be caused with an excess volume. Nine microlitres of NaOH (0.5 M) was found to be an appropriate volume for NaOH as this gave highest reaction intensity (Fig. 7a). The DSDM volume was increased from 1 to 18 μL. Initially this gave an increase in color up to a volume of 12 μL. As 12 μL was the minimum volume that gave the highest intensity response, it was chosen as the optimum DSDM volume (Fig. 7b). The optimum reaction time was found to be 30 min (Fig. 7c).

**Fig. 8:**
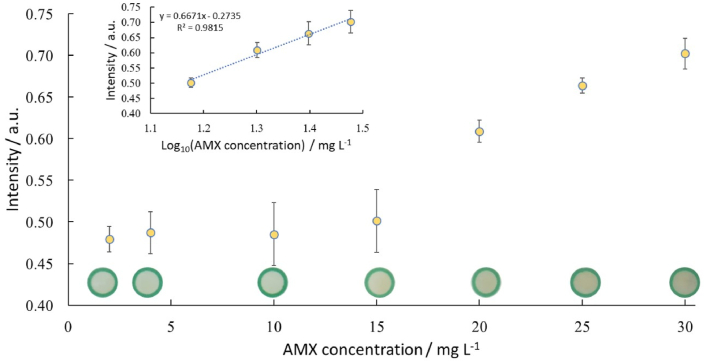
Calibration curve showing an increasing trend exceeding 10 mg L^−1^ for AMX (2–30 mg L^−1^), which had a logarithmic region between 10 and 30 mg L^−1^, n = 4.

Under the optimized parameters, using 12 μL of DSDM (10 mM) and 9 μL NaOH (0.5 M), a calibration curve over the range of 2–30 mg L^−1^ was obtained for AMX concentration determination (Fig. 8). The images were taken 30 min after AMX addition and data showed a plateau increase in intensities above 10 mg L^−1^. A fluctuation and fairly weak color intensities were observed at lower concentrations. Thus the paper-based microfluidic device is suitable for semi-quantitative determinations.

**Fig. 9:**
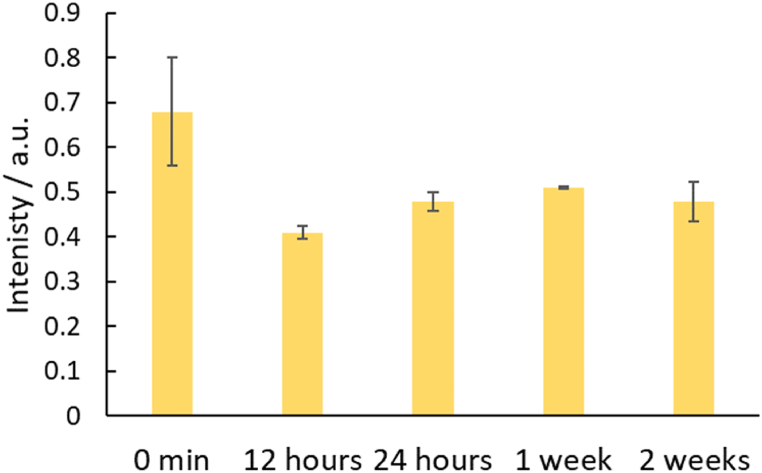
Stability experiments from the start of the reaction until two weeks. Storage was in ambient conditions. (n = 3).

Device stability was also investigated over a period of two weeks (Fig. 9). It was found that initially, the reaction was more intense forming a deeper yellow color due to the reagents being wet when the sample was added. However, after drying the reagents, the intensity decreased. There was a small increase in intensity between 12 and 24 h, but the reaction remained stable for two weeks suggesting reagents were not degrading when stored. This suggests devices have the potential to be stored and transported to needed locations for onsite testing.

### 3.3 Analysis of pharmaceutical dosage forms

**Fig. 10:**
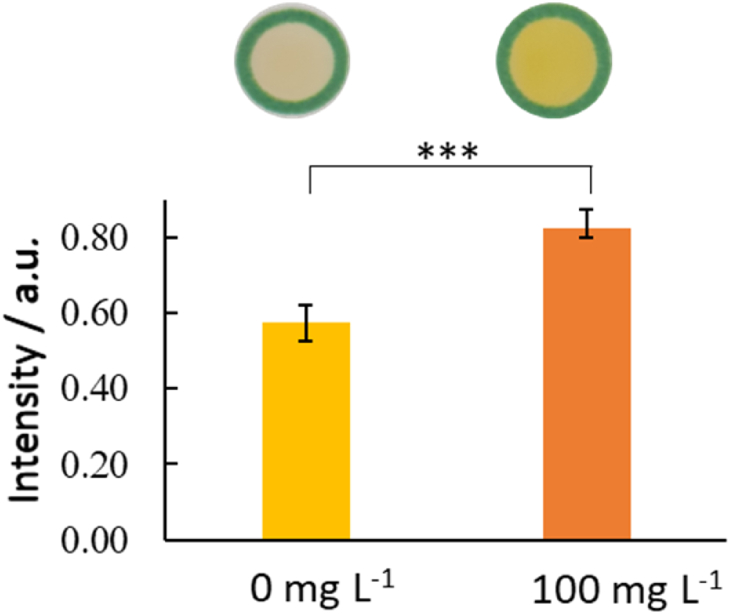
Comparison of a blank sample (negative control) and sample at 100 mg L^−1^ (positive control), showing significant difference between samples, n = 2 devices, 4 repeats per device. T-test assuming even variance, (f = 1.16 < F_crit_ = 3.79, t_stat_ = −10.5 < -t_crit two tail_ = -2.14, *p* = 4.90 × 10^−8^*).*

The applicability of the proposed method for the determination of AMX concentrations within a pharmaceutical product was investigated. The tested product was **Amoxicillin® injection** (500 mg amoxicillin sodium) as described in the experimental section. Two different concentrations (10 and 20 mg L^−1^) were prepared. Their absorbance was compared to the calibration curves. The results, as can be seen in Table 1, indicate a quite high precision and accuracy of the method. A similar series of experiments was completed using the microfluidic paper-based devices to study the presence of AMX at 100 mg L^−1^ showing that at this concentration the paper-based devices can be indeed to be employed to clearly show the presence of the drug in comparison to a blank sample (Fig. 10).

**Table 1:** Analysis of Amoxicillin® injection formulations via UV/vis absorption.

UV/vis Method	Taken Concentration (mg L^−1^)	Found Concentration after analysis (mg L^−1^), n = 4	Rec.%	RSD%
**Amoxicillin® injection**	10.00	9.8	98	1.36
	20.0	19.4	97	0.98

## 4 Conclusion

Our study involved a colorimetric diazotization reaction for quantitative measurement of AMX in solution and in pharmaceutical preparations using absorption spectrophotometry. A comparison was carried out with an inexpensive, more easily disposable, portable and simple-to-use paper-based microfluidic analytical platform that provides the advantages to pharmaceutical analysis in real-world setting as well as in research omitting the need for sophisticated procedures and instruments. In future work, we would include the analysis of different types of drugs, as well as reagent stability study over an extended period of time, the potential of pre-storing the reagents and investigating reaction products stability.

## Compliance and ethics statement

Review and approval by an ethics committee was not needed for this study because this work is related to pharmaceutical formulations. For the same reason, informed consent was not required.

## Funding statement

This work is self-funded.

## Additional information

No additional information is available for this paper.

## Data availability statement

All data supporting the results and conclusions of this study were included in the article.

## CRediT authorship contribution statement

**Jwan Oday:** Writing – review & editing, Writing – original draft, Validation, Resources, Methodology, Investigation, Conceptualization. **Hind Hadi:** Validation, Resources, Methodology, Formal analysis, Conceptualization. **Parween Hashem:** Resources, Investigation, Funding acquisition. **Samantha Richardson:** Writing – review & editing, Validation, Software, Formal analysis. **Alexander Iles:** Methodology, Funding acquisition. **Nicole Pamme:** Writing – review & editing, Visualization, Supervision, Resources.

## Declaration of competing interest

The authors declare that they have no known competing financial interests or personal relationships that could have appeared to influence the work reported in this paper.
